# Process optimization and characterization of levan from *Sporosarcina globispora* MTCC 4776

**DOI:** 10.1038/s41598-026-62960-y

**Published:** 2026-07-16

**Authors:** Shah Nisha, S. Balaji, N. Kannan

**Affiliations:** Manipal Institute of Technology, Manipal Academy of Higher Education, Manipal, Karnataka 576104 India

**Keywords:** Levan, Optimization, Exopolysaccharide, SDG3, NMR, FTIR, Biochemistry, Biotechnology, Chemistry, Microbiology

## Abstract

This study aims to explore *Sporosarcina globispora* MTCC 4776 to produce levan using submerged fermentation. The assessment of carbon and nitrogen sources revealed that a moderate concentration of sucrose and tryptone improved levan production. Eight of the independent variables, concentration of sucrose, tryptone, CaCl₂·2H₂O, K₂HPO₄, MgSO₄·7H₂O, % of inoculum, incubation time and agitation speed was assessed with twelve experimental runs through Plackett–Burman design. Concentration of sucrose, tryptone and agitation speed were found to be significant. These factors were further optimized using Central Composite Design, yielding a maximum of 1.41 g/L of levan, which is a 6.2-fold increase compared to unoptimized conditions. The extracted levan was characterized for its structure and physicochemical properties by FTIR, ^1^H/^13^C-NMR, TGA/DTG, DSC, and XRD. These results confirmed the presence of a β-(2 → 6)- linked fructofuranosyl chain with excellent thermal stability and a semi-crystalline structure. In the future, studies on the purification and application of levan will be conducted to assess the sample’s potential.

**Supplementary Information:**

The online version contains supplementary material available at 10.1038/s41598-026-62960-y.

## Introduction

Exopolysaccharides (EPS) are widely secreted by microorganisms into their external environment during growth and metabolism. They play a key role in maintaining the structural and functional integrity of the biofilms and influencing the physicochemical properties of microbial matrices^1,2^. The molecular weight of bacterial EPS usually ranges between 10 and 1000 kDa, with some polymers exceeding more than 2000 kDa, which is influenced by the organism producing them and the cultivation conditions^1^. Bacterial exopolysaccharides exhibit functional characteristics of biocompatibility, biodegradability, non-toxicity, gelation ability, etc., which have significantly advanced their applications across various domains^1^.

Bacterial exopolysaccharides can be categorized based on location, chemical composition, and functionality. Based on cell association, they are classified as capsular polysaccharides, which remain attached to the cell surface to form protective capsules, and slime polysaccharides, which are loosely attached or completely secreted into the extracellular environment. They are classified into homopolysaccharides and heteropolysaccharides based on their structure. Homopolysaccharides are α-d-glucans, β-d-glucans, fructans, and polygalactans. Heteropolysaccharides have repeating units of two or more different monosaccharides, including D-glucose, L-rhamnose, glucuronic acid, N-acetylglucosamine etc. with non-carbohydrate substituents such as acetyl, phosphate, and glycerol groups^3^.

Among EPS, microbial fructans, especially levan-type β-(2,6)-fructans, are increasingly recognized as prebiotic fibers and functional ingredients with attractive rheological and biological properties, encouraging the discovery of new producing strains and the development of efficient production processes^4^. Although research has been carried out on the production of EPS from different genera of bacteria, very few studies have examined EPS biosynthesis in *Sporosarcina* species. This bacterium, which belongs to the genus *Sporosarcina*, is closely related to the *Bacillus* species. It has mainly been studied with respect to microbially induced calcium carbonate precipitation and bio-cement formation^5^. However, recent work has been done regarding the possibility of their use in the synthesis of polymers. *Sporosarcina pasteurii* is mostly known for its urease activity and biogeochemistry with consistently high levels of urease and metabolism regulatory systems which may affect EPS synthesis^6^. *Sporosarcina psychrophila* MTCC2908 is one of the new bacteria isolated in recent research that can be used for production under specific culture conditions^7^. Studying polymer production in the genus *Sporosarcina* is a nascent field of research that may lead to the identification of unique biopolymers. Few studies have been conducted on *Sporosarcina* strains, necessitating further research. In this context, psychrophilic *Sporosarcina globispora* (formerly *Bacillus globisporus*) appears to be an interesting yet untapped source to produce levan, owing to its unique characteristics and genetic ability to metabolize carbohydrates.

The media composition, particularly carbon and nitrogen sources, strongly influences levan yield and structure. Sucrose and glucose are effective substrates, with sucrose preferred due to higher carbon conversion efficiency and its metabolism via glycolysis and the pentose phosphate pathway, generating precursors for levan biosynthesis (Peng et al., 2025; Xu et al., 2017). Nitrogen sources affect both growth and production, with organic sources outperforming inorganic ones due to their ability to supply essential nutrients and cofactors. A high C:N ratio enhances the production^10,11^. Sucrose-derived levan shows distinct properties, with molecular weights ranging from 3.789 × 10^4^ to > 10^6^ Da and generally higher yields of lower molecular weight polymers than glucose-derived EPS^9^. The structural diversity arises from glucan and fructan enzymes which produces polymers such as dextran and levan, with fructans characterized by β-(2 → 1) or β-(2 → 6) linkages. Functionally, plant fructans act as storage carbohydrates and stress protectants, whereas microbial fructans contribute to stress resistance, biofilm formation, and carbon storage^12–14^.

Levan production depends on the fermentation strategy, with submerged fermentation (SmF) widely used for its control, yield, and low risk of contamination^15,16^. Fed-batch improves yields by maintaining substrate balance and reducing viscosity issues, achieving 40–73% higher production than batch systems^17,18^. Solid-state fermentation (SSF) can yield 2–4.6 times more product in certain cases^19^.

In the present study, one of the fructan-type exopolysaccharides, Levan from *Sporosarcina globispora* MTCC 4776, was produced and optimized in a submerged fermentation process. The produced levan was subsequently purified and characterized to understand its structural and physicochemical properties. To our understanding and knowledge, this work represents the first report of levan production by *Sporosarcina globispora* MTCC 4776.

## Materials and methods

### Materials

All chemicals, reagents, and media components used in the study is of analytical grade and procured from Loba Chemie, Mumbai, India, Sigma-Aldrich, Bangalore, India, and HiMedia, Mumbai, India.

### Bacterial strain and preservation

*Sporosarcina globispora* MTCC 4776, a gram-positive, spore-forming bacterium, was procured from the Microbial Type Culture Collection (MTCC), Chandigarh, India. The culture was maintained on nutrient agar slants at 4 °C and periodic transfer of culture was performed to maintain the viability.

### Bacterial growth curve

For inoculum preparation, a single loopful of culture was aseptically transferred into 100 mL of sterile nutrient broth and incubated at 37 °C with agitation at 150 rpm. Growth was monitored by change in turbidity. Aliquots (2 ml) of the samples were withdrawn at every two hours interval for 30 h. The absorbance of the resuspended solution was measured at 600 nm using a UV–Vis spectrophotometer (GENESYS 180 spectrophotometer, Thermo Scientific). A time-course profile of organism was constructed, and different phases of microbial growth were observed. The mid-exponential phase of the microorganism was observed at 17 h and the same inoculum age is used as the inoculum for subsequent fermentation experiments.

### Qualitative assessment of EPS

Nutrient agar supplemented with 10% (w/v) sucrose was used for qualitative assessment. In short, the solidified agar plates were inoculated with the 0.5 µl of overnight culture and spread on the plate. The plate is incubated for 36 h at 37 °C. The change in appearance and mucoid colonies is indicative of EPS production^20^.

### Urease activity assay

Urease activity of *Sporosarcina globispora* MTCC 4776 was assessed on both solid and liquid media by applying protocol of the American Society for Microbiology (Christensen’s urea agar and Stuart’s urea broth)^21^. Briefly, an 18 h culture was inoculated into both agar slants and broth containing urea and phenol red. The test samples were incubated at 37 °C for 48 h. Production of urease was recorded qualitatively based on the development of a pink colour in the medium, compared with the yellow uninoculated controls (Supplementary Fig. S1a).

### Effect of media composition

Different media composition were prepared, medium A (g/L) consists of sucrose (100), yeast extract (2), MgSO₄·7H₂O (0.2), K₂HPO₄ (3), CaCl₂·2H₂O (2) and medium B (g/L) is made up of sucrose (200), NH₄Cl (60), K₂HPO₄ (11), MgSO₄·7H₂O (3), MnSO₄·H₂O (3), CaCl₂·2H₂O (2), FeCl₃ (2) to evaluate EPS production. All medium components were dissolved in distilled water and autoclaved at 121 °C for 15 min. Each 250 mL flask containing 100 mL of sterile medium was inoculated with 2% (v/v) inoculum. Fermentation was performed at 37 °C with shaking at 150 rpm for 24 h.

### Effect of carbon source

To evaluate the effects of different carbon sources and their combinations on levan production, a total of eleven distinct media formulations (M1–M11) were prepared (supplementary Table S1a). The base medium was derived from two formulations (Medium A & B). Media M1–M5 were designed using yeast extract as the nitrogen source (low-nitrogen condition). Media M6–M11 were formulated using ammonium chloride (NH₄Cl) as the nitrogen source (high nitrogen condition) along with additional trace minerals. Each medium was supplemented with different carbon sources (sucrose, glucose, or fructose) either individually or in various combinations. All components were dissolved in distilled water, sterilized by autoclaving at 121 °C for 15 min, and cooled before inoculation.

### Effect of nitrogen source

The organic (Yeast extract, Tryptone, Casein, Peptone) and inorganic (NH_4_Cl, (NH_4_)_2_SO_4_) nitrogen sources were individually added into the basal medium containing fixed sucrose, MgSO₄·7H₂O, K₂HPO₄, and CaCl₂·2H₂O (supplementary Table S1b). Each medium was inoculated with 5% of inoculum and incubated under shaking conditions at 37 °C for 24 h at 150 rpm.

### Screening of variables by Plackett–Burman design (PBD)

A statistical experimental model was created with JMP software to screen the parameters for levan production using PBD. The experimental design matrix of PBD with coded and uncoded levels as shown in Table 1.

**Table 1:** Coded and actual factor levels with experimental and predicted yield responses of PBD.

Run	X1	X2	X3	X4	X5	X6	X7	X8	Yield (experimental, g/L)	Yield (predicted, g/L)
1	30 (+)	0.4 (−)	0.4 (−)	0.2 (−)	0.05 (+)	8 (+)	34 (+)	150 (−)	1.08	0.93
2	30 (+)	0.5 (+)	0.5 (+)	0.2 (−)	0.05(+)	8 (+)	30 (−)	160 (+)	0.98	0.91
3	20 (−)	0.5 (+)	0.5 (+)	0.2 (−)	0.05(+)	5 (−)	30 (−)	150 (−)	0.8	0.87
4	30 (+)	0.5 (+)	0.4 (−)	0.4 (+)	0.05(+)	5 (−)	34 (+)	150 (−)	0.88	0.73
5	30 (+)	0.4 (−)	0.5 (+)	0.4 (+)	0.04 (−)	8 (+)	30 (−)	150 (−)	1.18	1.1
6	20 (−)	0.4 (−)	0.4 (−)	0.4 (+)	0.05(+)	8 (+)	30 (−)	160 (+)	0.69	0.68
7	20 (−)	0.5 (+)	0.5 (+)	0.4 (+)	0.04 (−)	8 (+)	34 (+)	150 (−)	0.81	0.88
8	20 (−)	0.4 (−)	0.5 (+)	0.4(+)	0.05 (+)	5 (−)	34 (+)	160 (+)	0.71	0.72
9	20 (−)	0.4 (−)	0.4 (−)	0.2 (−)	0.04 (−)	5 (−)	30 (−)	150 (−)	0.92	0.85
10	20 (−)	0.5 (+)	0.4 (−)	0.2 (−)	0.04 (−)	8 (+)	34 (+)	160 (+)	0.78	0.71
11	30 (+)	0.4 (−)	0.5 (+)	0.2 (−)	0.04 (−)	5 (−)	34 (+)	160 (+)	0.97	0.96
12	30 (+)	0.5 (+)	0.4 (−)	0.4(+)	0.04 (−)	5 (−)	30 (−)	160 (+)	0.74	0.71

X1-sucrose concentration (% w/v); X2-tryptone concentration (%w/v); X3-CaCl₂·2H₂O (% w/v); X4-K₂HPO₄ (% w/v); X5- MgSO₄·7H₂O (% w/v); X6-inoculum %; X7-time (h); X8-agitation speed (rpm)

### Optimization of the process parameters using central composite design

Through PBD, the prediction profiler identified sucrose concentration, agitation speed, and tryptone concentration as the most significant parameters. These three process parameters identified as the most significant parameters were consequently selected for further optimization using RSM based on CCD. The actual values used for parameters are shown in Table 2. CCD was used for the optimization of the key process parameters influencing the yield. The structure of CCD consists of 3 types of experimental points shown in Supplementary Table S1c. A statistical experimental model was designed with JMP software to optimize the parameters for exopolysaccharide production. Twenty experimental runs were constructed with six center points runs to estimate error and to ensure model reliability. The coded experimental design matrix of CCD is presented in Table 2. The experiments were carried out in 100 mL of fermentation medium.

**Table 2:** The experimental and predicted values of CCD with uncoded and coded combinations of parameters used for optimization.

Run	X1	X2	X3	Yield (experimental, g/L)	Yield (predicted, g/L)
1	30 (−1)	135 (−1)	0.3 (−1)	0.41	0.45
2	30 (−1)	135 (−1)	0.5 (+1)	0.74	0.7
3	30 (−1)	145 (+1)	0.3 (−1)	0.45	0.62
4	30 (−1)	145 (+1)	0.5 (+1)	0.78	0.8
5	40 (+1)	135 (−1)	0.3 (−1)	0.51	0.58
6	40 (+1)	135 (−1)	0.5 (+1)	0.79	0.71
7	40 (+1)	145 (+1)	0.3 (−1)	0.44	0.54
8	40 (+1)	145 (+1)	0.5 (+1)	0.57	0.62
9	25 (−2)	140 (0)	0.4 (0)	0.68	0.63
10	45 (+2)	140 (0)	0.4 (0)	0.61	0.61
11	35 (0)	130 (−2)	0.4 (0)	0.32	0.36
12	35 (0)	150 (+2)	0.4 (0)	0.61	0.51
13	35 (0)	140 (0)	0.2 (−2)	0.22	0.06
14	35 (0)	140 (0)	0.6 (+2)	0.31	0.37
15	35 (0)	140 (0)	0.4 (0)	1.22	1.31
16	35 (0)	140 (0)	0.4 (0)	1.41	1.31
17	35 (0)	140 (0)	0.4 (0)	1.35	1.31
18	35 (0)	140 (0)	0.4 (0)	1.29	1.31
19	35 (0)	140 (0)	0.4 (0)	1.32	1.31
20	35 (0)	140 (0)	0.4 (0)	1.41	1.31

X1- sucrose concentration (% w/v); X2- agitation speed (rpm); X3- tryptone concentration (%w/v)

The analysis of the experimental results was conducted using analysis of variance (ANOVA) to establish the significance and adequacy of the established quadratic model. The adequacy of the established model was evaluated using the coefficient of determination (R^2^), adjusted R^2^, and lack-of-fit test. All calculations were done using the JMP software.

### Validation

Validation of predicted optimal medium composition and conditions were investigated. The predicted media consist of (g/100 mL): Sucrose, 35; Tryptone, 0.4; MgSO₄·7H₂O, 0.04; K₂HPO₄, 0.2; and CaCl₂·2H₂O, 0.5, was inoculated with 8% inoculum, incubated at 140 rpm for 30 h. This experiment was carried out to ensure the accuracy and reliability of the developed model by comparing the experimental results with the predicted results.

### Extraction of levan

After fermentation, levan was recovered following the method described by^22^, with slight modifications. The cultures were centrifuged at 10,000 rpm for 15 min at 4 °C to remove biomass. The clear supernatant was mixed with two volumes of pre-chilled absolute ethanol (1:2 ratio) and kept at 4 °C overnight to precipitate the levan. The precipitate was recovered via centrifugation (10,000 rpm, 15 min), washed twice with distilled water, and centrifuged again. The final pellet was dried at 60 °C to obtain the constant mass and weighed. The samples were cryopreserved and was lyophilized to obtain dry powder. The dry mass was determined using an analytical balance and yield was expressed as g/L of culture by dividing the lyophilized mass by the initial working volume of 0.1 L.

### Characterization of extracted levan

The purified levan was collected and characterized to determine its structural and physicochemical properties.

#### Fourier Transform infrared spectroscopy (FTIR)

The functional groups in the sample were identified using Fourier transform infrared (FTIR) spectroscopy. The spectra were recorded using a Bruker Alpha II FTIR spectrophotometer operated in the Attenuated Total Reflectance (ATR) mode. Measurements were taken in the spectral range of 4000 cm^−1^ to 400 cm^−1^, with a resolution of 4 cm^−1^. Functional groups and other characteristic polysaccharide moieties were identified by comparison with reference spectra^23^.

##### Nuclear magnetic resonance (NMR) spectroscopy

Structural elucidation was performed using NMR (Bruker Ascend 400 MHz spectrometer). Approximately 10 mg of lyophilized sample was dissolved in 0.5 mL deuterium oxide (D₂O). The ^1^H and ^13^C NMR spectra were recorded.

##### X-ray diffraction (XRD)

The crystalline or amorphous nature of the product was determined using XRD (Rigaku Miniflex 600, 5th generation). Lyophilized powder was evenly spread onto the sample holder. The diffractometer operated at 40 kV and a current of 15 mA. The analysis was performed at room temperature in scan mode over a 2θ range of 0°–80° at a scan rate of 2°/min.

##### Thermal characterization of sample

The thermal stability and degradation behavior of the lyophilized sample were analyzed using a PerkinElmer STA 6000 thermal analysis system equipped with an autosampler and operated using the Pyris software. Approximately 5–10 mg of the sample was placed in an aluminum crucible, and an empty pan was used as the reference. Thermal analysis was carried out from 30 to 800 °C at a heating rate of 20 °C/min under a nitrogen atmosphere. TGA curves (weight loss vs. temperature) were plotted to assess the moisture content and thermal decomposition. DTG analysis (rate of weight loss vs. temperature) was used to identify the precise degradation peaks and assess the multistep decomposition behavior.

Thermal transitions of the sample, such as the glass transition temperature (Tg) and melting point (Tm), were determined using a PerkinElmer DSC 6000 system with the Pyris software. Approximately 5–10 mg of dried sample was sealed in an aluminum pan and scanned over the temperature range of 30–800 °C, with a heating rate of 20 °C/min, under a nitrogen atmosphere. The obtained thermograms were analyzed to interpret the energy changes corresponding to physical transitions.

## Results and discussion

### Growth curve

**Fig. 1:**
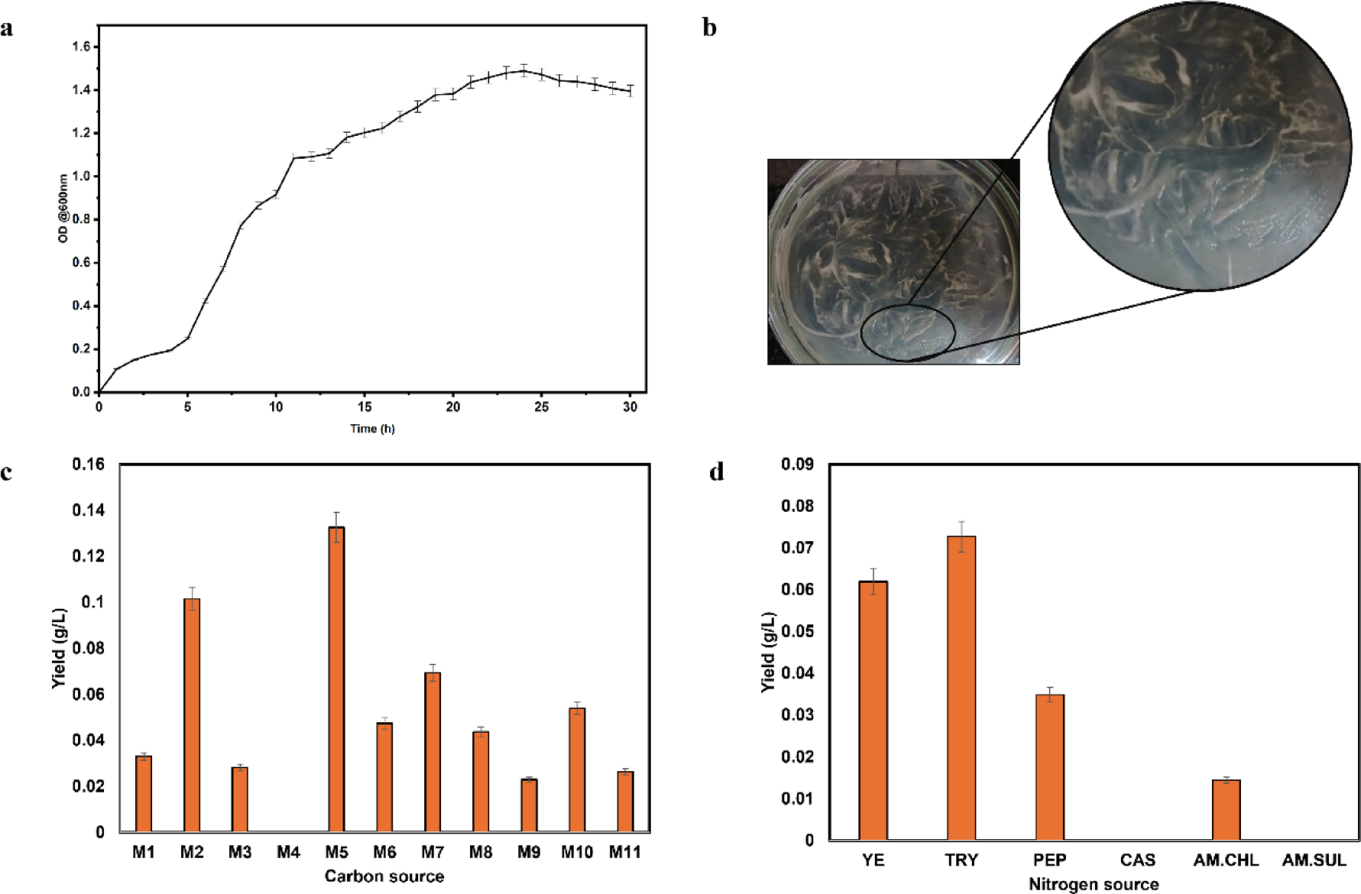
Screening of culture conditions and growth profile (**a**) Time course profile of *Sporosarcina globispora*, (**b**) agar plate assay showing mucoid colony formation, (**c**) effect of carbon source on production of fructan-type EPS, and (**d**) comparison of fructan-type EPS yield by varying the nitrogen source.

The growth of *Sporosarcina globispora* showed a typical pattern of batch growth (Fig. 1a). The lag phase at 0–4 h of cultivation, immediate exponential growth phase from 5 to 11 h, transition phase from 12 to 24 h, and a followed by stationary/early decline phase was observed. The observed growth pattern of *Sporosarcina globispora* is concurred with reported studies from^24^. The specific growth rate of the organism was 0.228 h^−1^, with a doubling time of 3.04 h. The metabolically active cultures at 17 h is considered and incubated into production media. It is observed that there is a high positive correlation between the incubation time and amount of biomass produced in the initial and mid-exponential growth phases, with subsequent reduction in growth rate may be due to aging of culture and the substrate depletion.

### Qualitative assessment

The mucoid appearance of the culture on sucrose-containing agar (Fig. 1b) can be explained by the action of enzyme levansucrase, which hydrolyses sucrose into glucose and fructose, transferring the fructosyl group of sucrose onto growing fructan chains. The hydrated fructan gel surrounding the cells increases their surface adhesion, microcolonies, and biofilm formation, giving the appearance of slimy growth. This is characteristic of levansucrase-positive bacteria growing on sucrose-rich media and offers qualitative evidence of the release of free levan into the medium. This is the qualitative confirmation of levan, which had good agreement with reported studies^25,26^. This may be confirmation that the microorganism is able to convert the soluble sucrose to levan. *S. globispora* MTCC 4776 exhibited positive urease activity. Both agar and broth represented the characteristic alkalinisation and colour change from yellow to pink compared with the control (Supplementary Fig. S1a), consistent with the urease-positive phenotype documented for several *Sporosarcina* species^5^.

### Carbon source

Sucrose, glucose, and fructose were identified and evaluated as carbon sources in eleven media (M1–M11) derived from two basic formulations, Media A and B (Supplementary Table S1a). Media A (M1–M5) contained a total sugar concentration of 10% (w/v), whereas Media B (M6–M11) contained 20% (w/v) sugar. In Media A, sucrose and its combinations were distributed as follows: M1 contained 10% sucrose only; M2 and M3 combined 10% sucrose with 5% glucose or 5% fructose, respectively; M4 contained 10% fructose alone; and M5 contained 10% glucose alone. In Media B, the total sugar load was doubled: M6–M8 contained 20% sucrose (alone or with 10% glucose or 10% fructose), while M9–M11 consisted of 20% fructose, 20% glucose, or a mixture of 20% glucose and 20% fructose, respectively. Levansucrase is a fructosyltransferase that exhibits high substrate specificity for sucrose, catalyzing the transfer of fructosyl units from sucrose to a growing β (2 → 6) fructan chain and releasing glucose as a by-product^27^. Media containing sucrose (M1–M3, M6–M8) are expected to favor levan synthesis, whereas media containing only glucose and/or fructose (M4, M5, M9–M11) are expected to primarily support biomass formation and may promote the production of other non-levan exopolysaccharides. The comparative yield obtained from media M1–M11 is summarised in Fig. 1c. Based on yield, the carbon-source media could be ranked as M5 > M2 > M10 > M7 > M8 > M6 > M11 > M3 > M1 > M9 > M4, indicating that formulations with glucose or mixed sugars under low-nitrogen conditions were generally superior to high-nitrogen, metal-enriched media. In our screening, M5 (10% glucose) yielded the highest apparent EPS titre; however, the product was highly hygroscopic, forming a sticky, cohesive mass upon drying. Such behaviour is consistent with the strong water-binding, film-forming, and hydrogel-forming properties of levan-type fructans and related bacterial EPS, which can lead to excessive moisture uptake and poor powder flowability. Hygroscopic product is undesirable for process development because it hampers efficient recovery, grinding, accurate weighing, and storage stability, and it can obscure intrinsic thermal and structural features during TGA/DSC and spectroscopic analyses.

Media B (M6–M11), all containing 20% (w/v) total sugar, produced black or charred nature of the product. The strong reduction in colour suggests that under conditions of high sugars, there have been extensive processes of non-enzymatic browning and caramelization of sucrose and fructose, causing destruction of the levan structure, and formation of coloured compounds with low molecular weight, like 5-hydroxymethylfurfural and other furan derivatives^28^. The presence of a high amount of sugar and amino groups from the nitrogen source, and some metals, might enhance the rate of the Maillard reaction and caramelization, forming mixed pigment complexes with different thermal and antioxidant characteristics and interfering with levan structure purity^29^. M1 (10% sucrose, no additional glucose or fructose) yielded a white, non‑hygroscopic product that could be dried into a stable powder and handled reproducibly. The exclusive presence of sucrose as the carbon source in M1 provides an optimal substrate for levansucrase‑mediated β-(2 → 6) levan synthesis^30^ while avoiding the excessive osmotic pressure and degradation phenomena seen at 20% sugar in Media B. Although its yield was lower than that of M5, the polymer from M1 exhibited superior physical stability and was taken further for optimization.

### Nitrogen source

M1–M4 Media contained organic nitrogen sources (yeast extract, tryptone, peptone, casein; each 0.2% w/v), whereas M5 and M6 contained inorganic nitrogen at comparatively high levels (6% w/v NH₄Cl or (NH₄) ₂SO₄). Out of the tested nitrogen sources, tryptone (M2) yielded the highest EPS production of 0.727 g/L, followed by yeast extract (M1, 0.619 g/L) and peptone (0.348 g/L). Casein did not produce a measurable yield under the conditions tested (Fig. 1d). In contrast, the inorganic nitrogen sources were less effective. NH_4_Cl (M5) yielded only 0.144 g/L, while (NH_4_)_2_SO_4_ failed to increase production. Organic nitrogen sources such as yeast extract, tryptone and peptone supply not only assimilable nitrogen but also amino acids, short peptides, vitamins and trace growth factors that support biomass formation and de-novo enzyme synthesis. For levan biosynthesis, this is particularly important because levansucrase is cell-associated enzyme whose production depends on active protein synthesis in a nutritionally rich environment. In the presence of yeast extract or tryptone (M1 and M2), the culture therefore receives a balanced supply of nitrogen and cofactors that favours both microbial growth and levansucrase expression^31^, leading to higher fructan-type EPS titres and structurally well-defined β-(2 → 6) fructan^32^. Although casein is an organic protein source, it is less readily solubilised and hydrolysed under the tested conditions, which may limit its contribution to rapid enzyme formation, and this was reflected in comparatively lower EPS production in M4.

In contrast, inorganic nitrogen salts in M5 and M6 (ṄḤ₄Cl and (NH₄)₂SO₄ at 6% w/v) provide ammonium as a simple nitrogen source. However, it lacks the support of amino acids, vitamins, and other complex nutrients. The high concentrations of nitrogen sources can shift metabolism towards biomass maintenance rather than secondary metabolite formation. Several studies have shown that the nature and level of nitrogen, and consequently the carbon-to-nitrogen (C/N) ratio, strongly influence both the amount and molecular size distribution of bacterial EPS: high inorganic nitrogen or low C/N typically favors smaller polymers and reduced EPS productivity, whereas moderate nitrogen and higher C/N promote synthesis of high-molecular-weight exopolysaccharides^33^. In our system, the poor performance of NH₄Cl and (NH₄)₂SO₄ is consistent with these observations and suggests that these inorganic sources did not adequately support levansucrase production or the biosynthesis of long-chain levan^25^.

### Initial screening of fermentation media using Plackett–Burman design

**Fig. 2:**
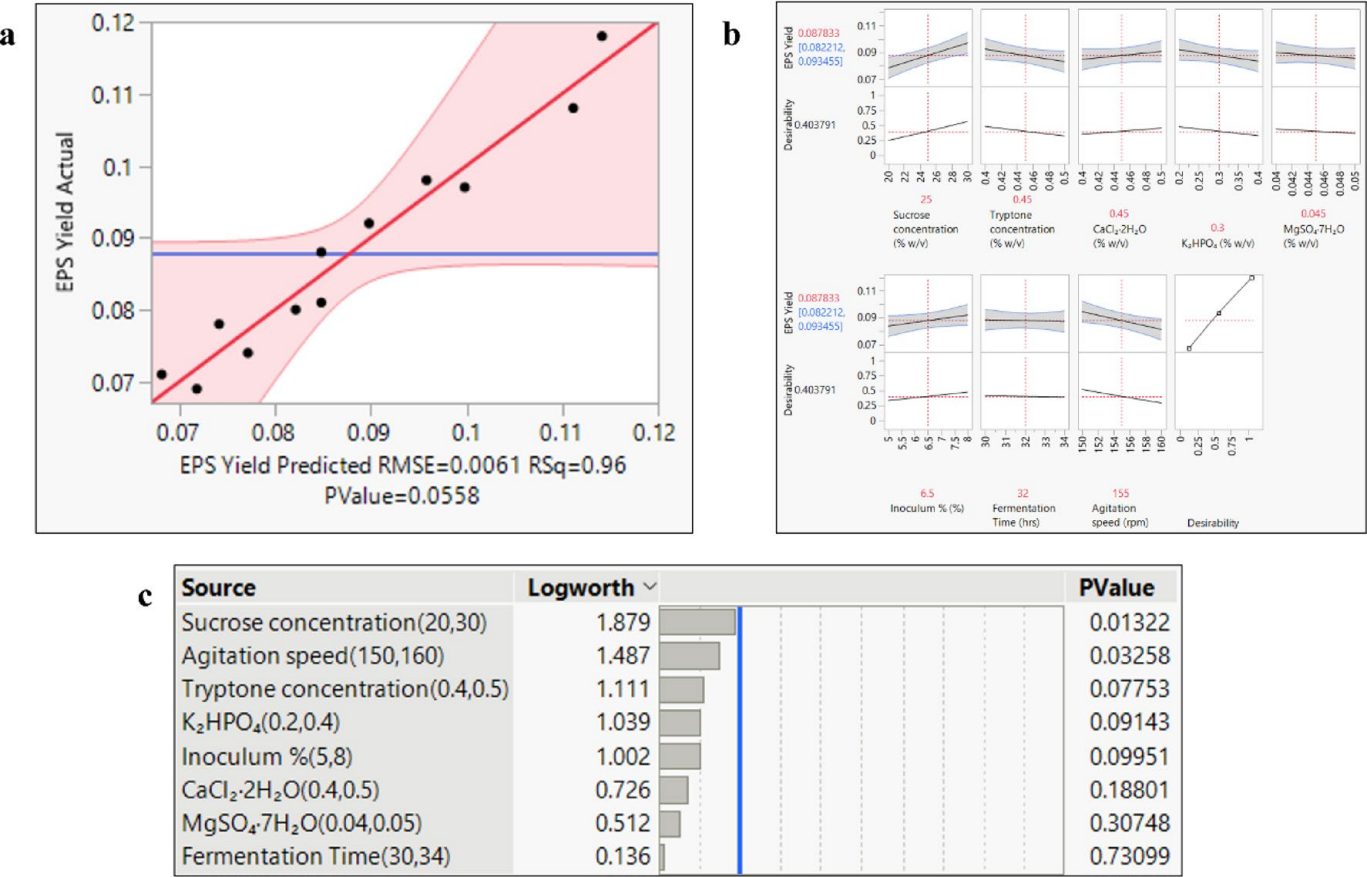
Plackett–Burman design (PBD) analysis for EPS (levan) production. (**a**) Normal plot showing correlation between actual and predicted EPS yield, (**b**) prediction profiler for EPS production, and (**c**) Pareto chart indicating significant factors influencing EPS production

The initial OVAT studies on the carbon and nitrogen sources indicated that the sucrose and complex organic nitrogen sources such as tryptone were conducive for levan production. However, the OVAT approach was not effective in accounting for the possible interactions that might occur among the various factors such as the concentration of sucrose, the speed of agitation, and the inoculum volume. In addition, the OVAT approach is inefficient in handling multiple variables and does not take into consideration the synergistic and antagonistic effects that might occur during the production of levan. Therefore, the sequential optimization design was employed. The strategy insights in the rapid differentiation of influential and non-influential factors. In the sequential optimization strategy, PBD was used to screen the parameters for yield. Eight factors such as concentration of sucrose, the concentration of tryptone, CaCl₂·2H₂O, K₂HPO₄, MgSO₄·7H₂O, inoculum level, incubation time, and agitation speed were screened and the most influencing factors on levan production were identified. The PBD matrix, including the number of experimental runs and the assignment of high (+) and low (−) levels for each factor, was generated using JMP software, which also provided tools for analysis. From the PBD design matrix (Table 1), it can be observed that the runs with high sucrose levels and high agitation levels (e.g., Runs 1, 5, 11) generally produced higher yield than those with low levels of sucrose and agitation (e.g., Runs 3, 6, 9); thus, there was a positive main effect of sucrose concentration and agitation on levan production. On the other hand, changes in the levels of mineral salts in the ranges studied had a limited influence on the yield, which implies that their effects are of secondary importance. The above trends (Fig. 2b), along with the regression coefficients, allow one to select the most important factors, namely, sucrose concentration, agitation speed, and tryptone concentration, to be considered in the CCD.

In PBD, the yield was ranged from 0.6 to 1.0 g/L, which indicates the significance of parameters selected for the screening of levan production. The model exhibited good predictability (Fig. 2a) with a coefficient of determination (R^2^) is 0.96, indicating that 96% of the variation of yield could be explained by the linear combination of the variables. The minimal residuals observed between experimental and predicted values confirm the adequacy of the model. The prediction profiler generated by JMP software screened three significant parameters such as sucrose concentration, agitation speed, and tryptone concentration^31^. The main effect of the independent variables was assessed. The variables sucrose concentration, CaCl₂·2H₂O and percentage of inoculum had positive effects whereas concentration of tryptone, K₂HPO₄, MgSO₄·7H₂O, agitation speed had negative effect on the response and time is found to be significant at the central point (Fig. 2b). PBD results indicated that the parameters influencing the production followed the order of significance: sucrose concentration > agitation speed > Tryptone > K_2_HPO_4_ > Inoculum > CaCl_2_·2H_2_O > MgSO_4_·7H_2_O > Time (Fig. 2c).

### Central composite design (CCD)

The significant parameters, concentration of sucrose, tryptone, and agitation speed, were further optimized using CCD at 5 levels with 20 different experimental runs. The coded CCD matrix is shown in Table 2. These results indicated that the yield increased from 0.22 g/L (Run 13) to 1.41 g/L (Run 16). The optimized medium containing 35 g/100 ml sucrose, 0.4 g/100 ml tryptone, and 140 rpm was found to produce a yield of 1.41 g/L, which is comparable with levan produced by bacteria so far reported in literature^34,35^. The results obtained from the experimental design matrix were fitted using a polynomial equation as a function of three coded parameters and are shown in Eq. 1.

$$\begin{aligned} {\mathrm{Y}} & \, = \, 0.{131}{-}0.00{\text{1 X}}_{{1}} + \, 0.00{\text{2 X}}_{{2}} + \, 0.00{\text{7 X}}_{{3}} {-}0.00{\text{4 X}}_{{1}} {\mathrm{X}}_{{2}} {-}0.00{\text{3 X}}_{{1}} {\mathrm{X}}_{{3}} {-}0.00{\text{1 X}}_{{2}} {\mathrm{X}}_{{3}} \\ & \quad {-}0.0{\text{18 X}}_{{1}}^{{2}} {-}0.0{\text{22 X}}_{{2}}^{{2}} {-}0.0{\text{27 X}}_{{3}}^{{2}} \\ \end{aligned}$$

where Y, EPS yield (g/L); X_1_, Sucrose concentration (g/100 mL); X_2_, Agitation speed (g/100 mL); X_3_, Tryptone concentration (g/100 mL).

**Fig. 3:**
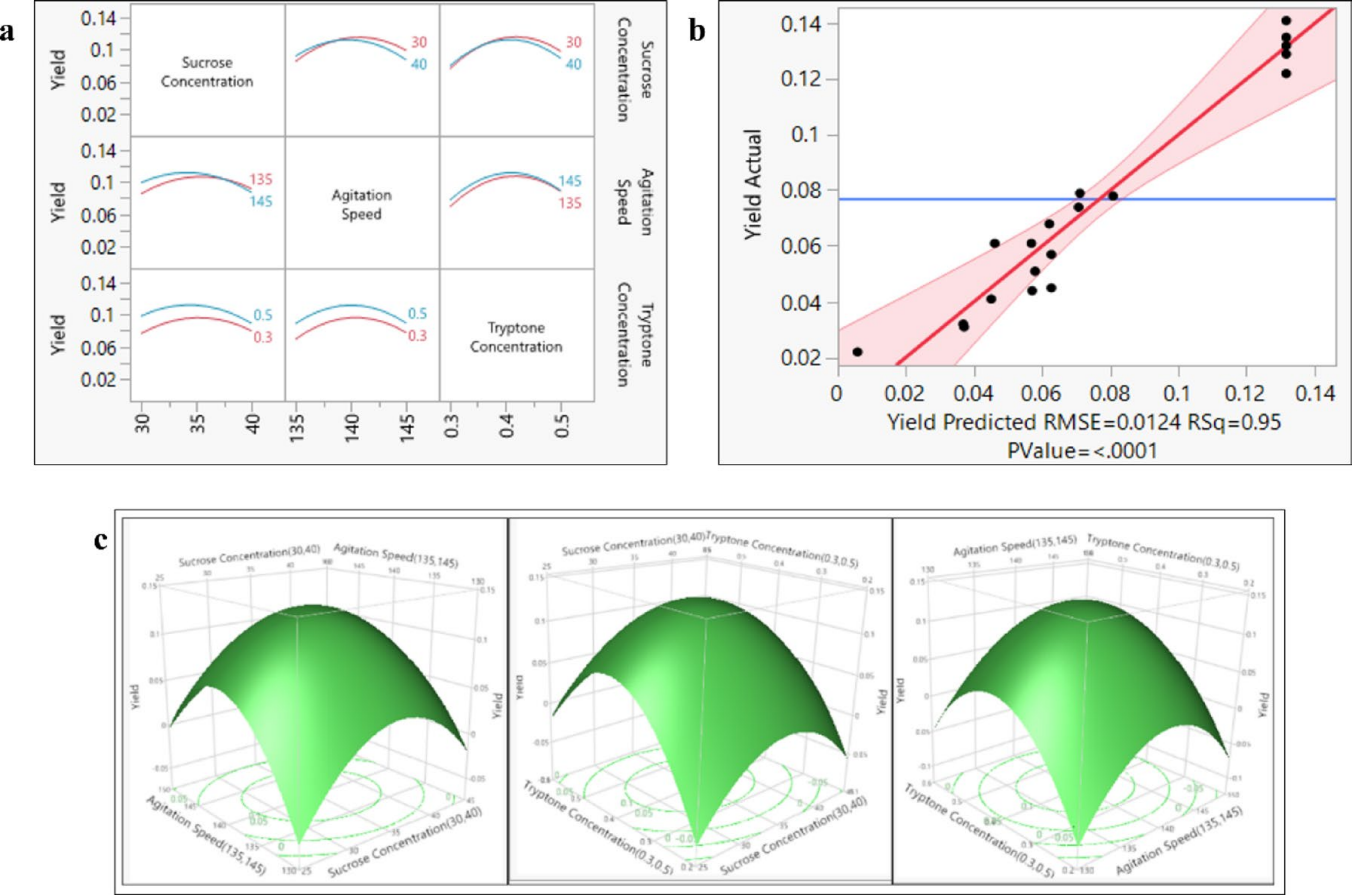
Central composite design (CCD) analysis for levan production (**a**) Interaction profiles of process parameters on levan yield, (**b**) correlation between experimental and predicted levan yield, and (**c**) response surface plots illustrating interaction effects of variables on levan yield.

From the optimization results (Fig. 3a), the concentration of sucrose was a major factor in production. For this medium, an optimal concentration of 35 g/100 mL was found to favor production, whereas lower and higher concentrations of sucrose in the range of 25–30 and 40–45 g/100 mL, respectively, resulted in reduced production. This indicated substrate inhibition at higher concentrations. This study on thermophilic species showed that cell growth decreased with increasing sucrose concentration, which agrees with previous reports^25,36^. In our study, both low and high levels of sucrose/tryptone led to reduced biomass and levan titers, indicating cellular stress resulting from an imbalance in carbon and nitrogen availability. At low sucrose concentrations, the reduced levan yield may be attributed to limited substrate availability, as insufficient sucrose can restrict both growth and fructan formation. In contrast, high sucrose concentrations may affect the osmolarity of the medium and result in substrate inhibition of growth and levan production, as documented by^37,38^. Under the optimized CCD condition, the maximum levan concentration of 1.41 g/L at an initial sucrose concentration of 350 g/L corresponded to an overall yield of approximately 0.004 g levan per g sucrose. At lower and higher sucrose concentrations of 250 g/L and 400 g/L, respectively, the yield was 0.002 and 0.001 g levan per g sucrose. This was calculated as Y_P/S_ = C_P_/C_S_, where C_P_ is the levan concentration (g/L), and C_S_ is the initial sucrose concentration (g/L). This calculation is consistent with previous levan studies that report yields in g levan per g sucrose^39,40^. Since this is based on the sucrose supplied rather than sucrose consumed, it should be interpreted as an apparent substrate yield rather than a true conversion yield.

Tryptone concentration of 0.4 g/100 mL was optimum, whereas reduced production was obtained at lower and higher concentrations of tryptone in the range of 0.2 and 0.6 g/100 mL, respectively. This indicated that excessive nitrogen favored biomass formation over levan. The higher concentration of nitrogen may be attributed to the release of crude urease enzyme, which may reduce the levan yield (Supplementary Fig. S1a). A similar response is observed in levels of agitation speed, with an optimal rate of 140 rpm. Agitation speeds of 130 and 150 rpm, respectively, reduced levan production.

The analysis of the model showed high statistical significance with *p* < 0.0001, and the coefficient of determination is high (R^2^ = 0.95), as shown in Fig. 3b. This indicates a strong correlation between the experimental and predicted values. The low RMSE value (0.0124) also indicated high model accuracy and reliability. The presence of overlapped curve (Fig. 3c) in the response surface plots clearly indicates that interaction between the variables exists. The response surface plots may identify the optimum level of independent variables to attain the maximum response. A significant interaction between the variables was observed. The adequacy of the model was significant as confirmed with experimental results closely matched the predicted values. The F-value and p-value were used to assess the significance of the terms in the established model. The terms that had p-values less than 0.05 were considered significant. The ANOVA results of the statistical analysis carried out in this study for levan production are given in Table 3. The model had a large F-value (F = 21.581, *p* < 0.0001), which revealed that the model is significant. The value of R^2^ (R^2^ = 0.99) indicates that the model fits well and has good explanatory and prediction capabilities. The *p* values < 0.05 show that the terms of the model are significant. In this study, significant linear, interaction, and quadratic terms were selected, while some terms were found to be insignificant (*p* > 0.05). The lack-of-fit test was non-significant (*p* = 0.0564), indicating that the model is a good representation of the experimental data without systematic error. The results confirm that the developed quadratic model is appropriate for predicting and optimizing fructan-type EPS production. The comparative analysis of levan yield with the previously reported literature (Supplementary Table S2a) from *Bacillus* sp. EPS003 (0.939 g/L), *Bacillus tequilensis* (1.84 g/L), and *Paenibacillus* sp. #210 (1.45 g/L), indicating that the present strain performs competitively as the best producer of levan^34,35,41^.

**Table 3:** ANOVA of CCD experiments for fructan-type EPS.

Source	*df*	Sum of squares	Contribution (%)	F ratio	Prob > F
Model	9	0.03002	94.85	21.581	< 0.0001*
Sucrose (X₁)	1	0.00002	0.07	0.1783	0.6818
Agitation (X₂)	1	0.00008	0.21	0.5536	0.474
Tryptone (X₃)	1	0.00098	2.36	6.318	0.0307
X₁X₂	1	0.00017	0.41	1.1071	0.3175
X₁X₃	1	0.00008	0.19	0.5054	0.4934
X₂X₃	1	0.00003	0.07	0.182	0.6787
X₁^2^	1	0.0082	19.76	52.9035	< 0.0001*
X₂^2^	1	0.0128	30.86	82.5993	< 0.0001*
X₃^2^	1	0.0190	46.07	123.3212	< 0.0001*
Lack of fit	5	0.0013	–	4.7389	0.0564
Pure error	5	0.0003	–	–	–
Total error	10	0.0015	–	–	–
C. total	19	0.0316	100	–	–

### Characterization of levan

The purified levan was assessed for its structural and physicochemical properties using the selected analytical methods.

#### FT-IR

**Fig. 4:**
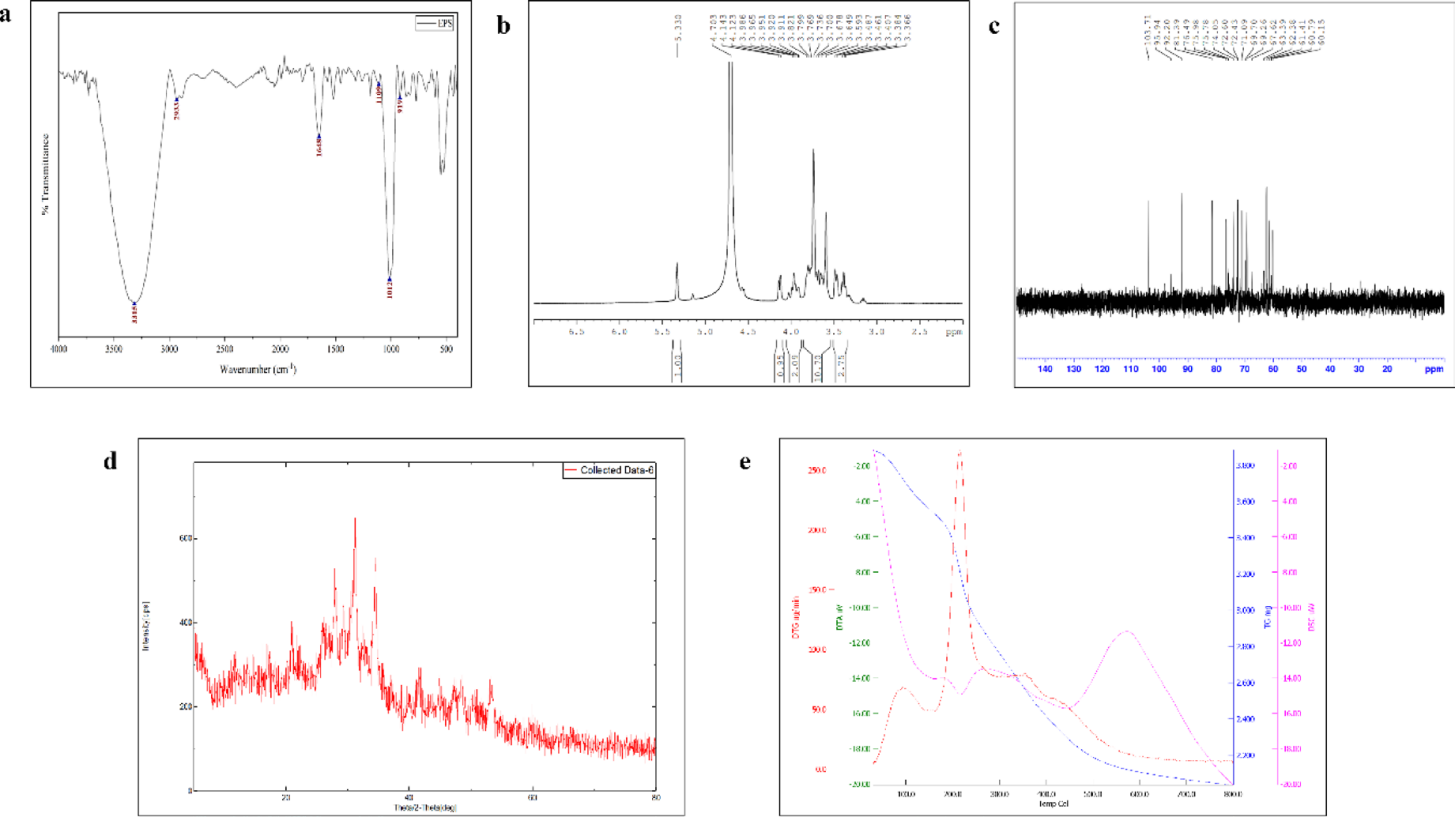
Characterization of purified levan produced by *Sporosarcina globispora* (**a**) FTIR spectrum (**b**) ^1^H NMR spectrum (**c**) ^13^C NMR spectrum (**d**) XRD pattern (**e**) TGA–DTG–DTA thermograms.

The FTIR analysis (Fig. 4a) of the extracted product showed absorption bands that were characteristic of a polysaccharide nature. A broad absorption band at 3200–3500 cm^−1^ corresponded to O–H stretching vibrations, indicating hydroxyl-rich polysaccharide chains. Peaks observed in the region of 1000–1200 cm^−1^ were attributed to C–O and C–O–C stretching vibrations, confirming glycosidic linkages. The absorption bands in the range of 923–809 cm^−1^ were consistent with furanose ring vibrations, confirming the presence of fructose units. The spectral agreement with the literature (Supplementary Table S2b) strongly suggests that the polymer produced is a fructan^23,42,43^.

#### NMR

The structural confirmation of the product was performed using ^1^H and ^13^C NMR spectroscopy. It provided signals consistent with polysaccharide structures. The ^1^H NMR spectrum displayed several peaks in the 3.3–4.2 ppm range corresponds to the ring protons of fructosyl units. This confirms the polysaccharide nature of the sample. Signals around 4.1 ppm are assigned to protons adjacent to glycosidic linkages. The absence of peaks in the aromatic and aldehyde regions further supports the carbohydrate nature of the polymer. A comparative analysis of this study with previous reports is provided in (Supplementary Table S2c)^23,42,43^.

The ^13^C NMR spectrum showed major peaks in the 60–105 ppm range, consistent with the polymer’s carbohydrate structure. The signals at 60–63 ppm were assigned to C6 carbons, while peaks between 67 and 76 ppm correspond to C3–C5 ring carbons of fructofuranose units. The chemical shifts of 103 ppm (C2) and 63.39 ppm (C6) correspond to the presence of β-(2 → 6) glycosidic linkages which are characteristic of levan. A detailed comparison with literature data is presented in Table 4.

**Table 4:** Comparison between ^13^C NMR chemical shift values of levan produced by *Sporosarcina globispora* MTCC 4776, and other bacteria.

Carbon atom	*Sporosarcina globispora* MTCC 4776	*Bacillus subtilis* MTCC 441	*Bacillus subtilis* ZW019	*Pantoea agglomerans* ZMR7
C1	60.15	59.97	59.88	60.58
C2	103.71	104.26	104.19	104.38
C3	76.49	76.36	76.28	75.83
C4	75.78	75.26	75.18	76.74
C5	81.39	80.34	80.27	80.35
C6	63.39	63.44	63.36	63.15
References	This study	^39^	^42^	^37^

#### XRD

The XRD pattern of levan produced from *Sporosarcina globispora* MTCC-4776 revealed a semi-crystalline nature. This is shown by the presence of both broad and sharp diffraction peaks over the scanned 2θ range (2°–80°), as shown in Fig. 4d. A sharp and intense peak at 34.46°, along with minor peaks at 31.29°, 32.74°, 47.32°, and 53.21°, indicated the presence of ordered crystalline domains. The broad regions between the sharp peaks are indicative of amorphous structures, which are commonly attributed to the randomly arranged polymer chains.

#### TGA–DTG–DSC

The TGA–DTG–DSC thermogram (Fig. 4e) of the produced levan shows a multi-stage thermal degradation pattern. The initial stage observed below 150 °C showed a weight loss in the TGA curve, which can be attributed to the removal of bound moisture present in the polysaccharide structure. A DTG peak at 100–120 °C supports this stage, indicating a low rate of weight loss, and an endothermic peak on the DTA curve indicates dehydration of the sample. The second stage of degradation occurred between 200 and 300 °C, during which a considerable mass loss was observed in the TGA curve. In this region, the DTG curve showed a sharp peak at 220 °C, indicating the maximum rate of thermal degradation due to cleavage of glycosidic bonds and depolymerization of levan backbones. The DTA curve also showed thermal transitions in this region, proving that structural breakdown has taken place in the polymer chain. In the third stage, which occurred from 300–600 °C, the TGA graph showed weight loss due to decomposition. This was also confirmed by the broad peak in the DTG graph. In the DTA graph, an exothermic peak was observed at 550–600 °C, which could have resulted from the oxidation of the char formed from the previous stages of decomposition. After 600 °C, the sample was losing weight very slightly, suggesting that it was undergoing the formation of stable char. The thermogram suggests that the levan produced was of moderate stability, as is the case with microbial exopolysaccharides.

## Conclusion

The strain *Sporosarcina globispora* MTCC4776 is a promising microbial source for levan production. It has been established through systematic media optimization with different combinations of carbon and nitrogen sources that sucrose-containing media induce levan biosynthesis, whereas media with higher concentrations of glucose and/or fructose are more likely to reduce or modify the product. The two-step optimization strategy yielded 1.41 g/L of levan from S. globispora MTCC 4776, which was 6.2-fold higher than the non-optimized medium (0.23 g/L). The obtained EPS was identified as levan through characterization studies. FTIR and NMR analyses showed that the backbone contained fructofuranosyl residues, whereas thermal stability and semi-crystallinity were determined using TGA/DSC and XRD. These results collectively outline a rationale, statistics-driven approach to designing a suitable medium and optimizing conditions for the synthesis of levan using *S. globispora*. The current optimized parameters and conditions can be used for scaling up and application-oriented studies in the future.

## Supplementary Information

Below is the link to the electronic supplementary material.

Supplementary Material 1

Supplementary Material 2

## Data Availability

All data supporting the findings of this study are available within the paper and its Supplementary Information.

## References

[1] Nwodo, U. U., Green, E. & Okoh, A. I. Bacterial exopolysaccharides: Functionality and prospects. *Int. J. Mol. Sci.* **13**, 14002–14015. 10.3390/ijms131114002 (2012). 23203046 10.3390/ijms131114002 PMC3509562

[2] Netrusov, A. I. et al. Exopolysaccharides producing bacteria: A review. *Microorganisms*10.3390/microorganisms11061541 (2023). 37375041 10.3390/microorganisms11061541 PMC10304161

[3] Ruas-Madiedo, P., Hugenholtz, J. & Zoon, P. An overview of the functionality of exopolysaccharides produced by lactic acid bacteria. *Int. Dairy J.*10.1016/S0958-6946(01)00160-1 (2002).

[4] Öner, E. T., Hernández, L. & Combie, J. Review of levan polysaccharide: From a century of past experiences to future prospects. *Biotechnol. Adv.* **34**, 827–844. 10.1016/j.biotechadv.2016.05.002 (2016). 27178733 10.1016/j.biotechadv.2016.05.002

[5] Carter, M. S. et al. Microbially induced calcium carbonate precipitation by *Sporosarcina pasteurii*: A case study in optimizing biological CaCO3 precipitation. *Appl. Environ. Microbiol.*10.1128/aem.01794-22 (2023). 37439668 10.1128/aem.01794-22 PMC10467343

[6] Ghosh, T., Bhaduri, S., Montemagno, C. & Kumar, A. *Sporosarcina pasteurii* can form nanoscale calcium carbonate crystals on cell surface. *PLoS ONE*10.1371/journal.pone.0210339 (2019). 30699142 10.1371/journal.pone.0210339 PMC6353136

[7] Biswas, B., Selvaraj, S. & Natarajan, K. Enhanced production and structural characterization of exopolysaccharide from *Sporocarcina psychrophile* MTCC 2908 through two step optimization for therapeutic evaluation. *Sci. Rep.*10.1038/s41598-025-10392-5 (2025). 40670478 10.1038/s41598-025-10392-5 PMC12267618

[8] Xu, C. et al. Effect of carbon source on production, characterization and bioactivity of exopolysaccharide produced by *Phellinus vaninii* Ljup. *An. Acad. Bras. Cienc.* **89**, 2033–2041 (2017). 29044312 10.1590/0001-3765201720150786

[9] Peng, Q. et al. Mechanisms of exopolysaccharide synthesis by *Streptococcus thermophilus* IMAU20246 determined by the carbon source. *Food Chem.*10.1016/j.foodchem.2025.144069 (2025). 40157104 10.1016/j.foodchem.2025.144069

[10] Biswas, J. & Paul, A. K. Optimization of factors influencing exopolysaccharide production by *halomonas xianhensis* sur308 under batch culture. *AIMS Microbiol.* **3**, 564–579 (2017). 31294176 10.3934/microbiol.2017.3.564 PMC6604991

[11] Berekaa, M. M. & Ezzeldin, M. F. Exopolysaccharide from *bacillus mojavensis* DAS10-1; production and characterization. *J. Pure Appl. Microbiol.* **12**, 633–640 (2018).

[12] Bounaix, M. S. et al. Biodiversity of exopolysaccharides produced from sucrose by sourdough lactic acid bacteria. *J. Agric. Food Chem.* **57**, 10889–10897 (2009). 19848387 10.1021/jf902068t

[13] Bruni, G. O., Qi, Y., Terrell, E., Dupre, R. A. & Mattison, C. P. Characterization of levan fructan produced by a *Gluconobacter japonicus* strain isolated from a sugarcane processing facility. *Microorganisms*10.3390/microorganisms12010107 (2024). 38257935 10.3390/microorganisms12010107 PMC10819292

[14] Franken, J., Brandt, B. A., Tai, S. L. & Bauer, F. F. Biosynthesis of levan, a bacterial extracellular polysaccharide, in the yeast *Saccharomyces cerevisiae*. *PLoS ONE*10.1371/journal.pone.0077499 (2013). 24147008 10.1371/journal.pone.0077499 PMC3795680

[15] Champagne, C. P., Gardner, N. J. & Lacroix, C. Fermentation technologies for the production of exopolysaccharide-synthesizing *Lactobacillus rhamnosus* concentrated cultures. *Electron. J. Biotechnol.* **10**, 211–220 (2007).

[16] Dávila Giraldo, L. R., Villanueva Baez, P. X., Zambrano Forero, C. J. & Arango, W. M. Production, extraction, and solubilization of exopolysaccharides using submerged cultures of agaricomycetes. *Bio. Protoc.* **13**, 1-11 (2023). 10.21769/BioProtoc.4841 PMC10560690 37817899

[17] Pan, L. et al. Pilot-scale production of exopolysaccharide from *Leuconostoc pseudomesenteroides* XG5 and its application in set yogurt. *J. Dairy Sci.* **105**, 1072–1083 (2022). 34998545 10.3168/jds.2021-20997

[18] Khani, M., Bahrami, A., Chegeni, A., Ghafari, M. D. & Zadeh, A. M. Optimization of carbon and nitrogen sources for extracellular polymeric substances production by *Chryseobacterium indologenes* MUT.2. *Iran. J. Biotechnol.* **14**, 13–18 (2016). 28959321 10.15171/ijb.1266 PMC5435027

[19] Seesuriyachan, P. et al. Optimization of exopolysaccharide overproduction by *Lactobacillus confusus* in solid state fermentation under high salinity stress. *Biosci. Biotechnol. Biochem.* **76**, 912–917 (2012). 22738958 10.1271/bbb.110905

[20] Korany, S. M., El-Hendawy, H. H., Sonbol, H. & Hamada, M. A. Partial characterization of levan polymer from *Pseudomonas fluorescens* with significant cytotoxic and antioxidant activity. *Saudi J. Biol. Sci.* **28**, 6679–6689 (2021). 34764781 10.1016/j.sjbs.2021.08.008 PMC8568983

[21] Urease Test Protocol. www.asmscience.org (2019).

[22] Sahyoun, A. M., Wong Min, M., Xu, K., George, S. & Karboune, S. Characterization of levans produced by levansucrases from *Bacillus amyloliquefaciens* and *Gluconobacter oxydans*: Structural, techno-functional, and anti-inflammatory properties. *Carbohydr. Polym.*10.1016/j.carbpol.2023.121332 (2024). 37940238 10.1016/j.carbpol.2023.121332

[23] Thakham, N. *et al.* Structural characterization of functional ingredient levan synthesized by *Bacillus siamensis* isolated from traditional fermented food in Thailand. *Int. J. Food Sci.* **2020, **1-12 (2020).

[24] Priyodip, P. & Balaji, S. An in vitro chicken gut model for the assessment of phytase producing bacteria. *3 Biotech*10.1007/s13205-019-1825-2 (2019). 31297307 10.1007/s13205-019-1825-2 PMC6614281

[25] Belghith, K. S., Dahech, I., Belghith, H. & Mejdoub, H. Microbial production of levansucrase for synthesis of fructooligosaccharides and levan. *Int. J. Biol. Macromol.* **50**, 451–458 (2012). 22234294 10.1016/j.ijbiomac.2011.12.033

[26] Ghazi, I. et al. Purification and kinetic characterization of a fructosyltransferase from *Aspergillus aculeatus*. *J. Biotechnol.* **128**, 204–211 (2007). 17056145 10.1016/j.jbiotec.2006.09.017

[27] Méndez-Lorenzo, L. et al. Intrinsic levanase activity of *Bacillus subtilis* 168 levansucrase (SacB). *PLoS ONE*10.1371/journal.pone.0143394 (2015). 26600431 10.1371/journal.pone.0143394 PMC4658133

[28] Chan, A. C., Mokhtar, S. U. & Hong, P. K. A systematic review on the determination and analytical methods for furanic compounds in caramel models. *J. Food Sci. Technol.* **62**, 1999–2012. 10.1007/s13197-025-06419-4 (2025). 41041498 10.1007/s13197-025-06419-4 PMC12484514

[29] Şen, D. & Gökmen, V. Kinetic modeling of Maillard and caramelization reactions in sucrose-rich and low moisture foods applied for roasted nuts and seeds. *Food Chem.*10.1016/j.foodchem.2022.133583 (2022). 35802969 10.1016/j.foodchem.2022.133583

[30] Raga-Carbajal, E., López-Munguía, A., Alvarez, L. & Olvera, C. Understanding the transfer reaction network behind the non-processive synthesis of low molecular weight levan catalyzed by *Bacillus subtilis* levansucrase. *Sci. Rep.*10.1038/s41598-018-32872-7 (2018). 30301900 10.1038/s41598-018-32872-7 PMC6177408

[31] Bouallegue, A. et al. Statistical optimization of levan: Influence of the parameter on levan structure and angiotensin I-converting enzyme inhibitory. *Int. J. Biol. Macromol.* **158**, 945–952 (2020). 32360961 10.1016/j.ijbiomac.2020.04.232

[32] Silbir, S., Dagbagli, S., Yegin, S., Baysal, T. & Goksungur, Y. Levan production by *Zymomonas mobilis* in batch and continuous fermentation systems. *Carbohydr. Polym.* **99**, 454–461 (2014). 24274530 10.1016/j.carbpol.2013.08.031

[33] Degeest, B. & De Vuyst, L. Indication that the nitrogen source influences both amount and size of exopolysaccharides produced by Streptococcus thermophilus LY03 and modelling of the bacterial growth and exopolysaccharide production in a complex medium. *Appl. Environ. Microbiol.*10.1128/aem.65.7.2863-2870.1999 (1999). 10388677 10.1128/aem.65.7.2863-2870.1999 PMC91430

[34] Marimuthu, S. & Rajendran, K. Structural and functional characterization of exopolysaccharide produced by a novel isolate *Bacillus* sp. EPS003. *Appl. Biochem. Biotechnol.* **195**, 4583–4601 (2023). 36705841 10.1007/s12010-023-04368-2

[35] Kumaresan, P., Purayil, J. N. & Preethi, K. Valorization of pineapple (*Ananas comosus*) peel waste for levan production: Assessment of biological activities. *Int. J. Biol. Macromol.*10.1016/j.ijbiomac.2025.139482 (2025). 39778827 10.1016/j.ijbiomac.2025.139482

[36] Phengnoi, P., Teerakulkittipong, N., Teeparuksapun, K., Lirio, G. A. & Jangiam, W. Optimization of levan biosynthesis from *Bacillus siamensis* using batch and continuous stirred-tank bioreactors: A response surface methodology approach. *Biotechnol. Rep.*10.1016/j.btre.2025.e00908 (2025). 10.1016/j.btre.2025.e00908 PMC12344194 40808893

[37] Al-Qaysi, S. A. S. et al. Bioactive levan-Type exopolysaccharide produced by *Pantoea agglomerans* ZMR7: Characterization and optimization for enhanced production. *J. Microbiol. Biotechnol.* **31**, 696–704 (2021). 33820887 10.4014/jmb.2101.01025 PMC9705920

[38] González-Garcinuño, Á., Tabernero, A., Sánchez-Álvarez, J. M., Galán, M. A. & Martin del Valle, E. M. Effect of bacteria type and sucrose concentration on levan yield and its molecular weight. *Microb. Cell Fact.* **16**, 1-11 (2017). 10.1186/s12934-017-0703-z PMC5442672 28535808

[39] Veerapandian, B. et al. Production and characterization of microbial levan using sugarcane (*Saccharum* spp.) juice and chicken feather peptone as a low-cost alternate medium. *Heliyon*10.1016/j.heliyon.2023.e17424 (2023). 37484316 10.1016/j.heliyon.2023.e17424 PMC10361384

[40] Chidambaram, J. S. C. A. et al. Studies on solvent precipitation of levan synthesized using *Bacillus subtilis* MTCC 441. *Heliyon*10.1016/j.heliyon.2019.e02414 (2019). 31687543 10.1016/j.heliyon.2019.e02414 PMC6819800

[41] Mendonça, C. M. N. et al. Characterization of levan produced by a *Paenibacillus* sp. isolated from Brazilian crude oil. *Int. J. Biol. Macromol.* **186**, 788–799 (2021). 34245738 10.1016/j.ijbiomac.2021.07.036

[42] Wang, J. et al. Biosynthesis and structural characterization of levan by a recombinant levansucrase from *Bacillus subtilis* ZW019. *Waste Biomass Valoriz.* **13**, 4599–4609 (2022).

[43] Yu, X. et al. Structural analysis of macromolecular levan produced by *Bacillus megaterium* GJT321 based on enzymatic method. *Int. J. Biol. Macromol.* **93**, 1080–1089 (2016). 27670726 10.1016/j.ijbiomac.2016.09.086

