# Supplementary material for: Process optimization and characterization of levan from Sporosarcina globispora MTCC 4776
Source: Sci Rep. 2026 Jul 16;16:25704. doi: 10.1038/s41598-026-62960-y (PMC13482570; doi:10.1038/s41598-026-62960-y)
Supplement: Supplementary file 1 [file 41598_2026_62960_MOESM1_ESM.docx]

Table S2a Comparison of microbial species, cultivation conditions, and levan yields

| Microorganism | Conditions | Yield | Reference |
| --- | --- | --- | --- |
| *Sporosarcina globispora* MTCC 4776 | Sucrose: 350 g/L  Temperature: 37°C  Agitation speed: 140 rpm  Time: 34 hours | 1.41 | This study |
| *Bacillus sp.* EPS003 | Sucrose: NA  Temperature: 37°C  Agitation speed: 120–160 rpm  Time: 48–72 hours | 0.939 | [33] |
| *Bacillus tequilensis* | Sucrose: 200 g/L  Temperature: 37°C  Agitation speed: NA  Time: 48 hours | 1.84 | [34] |
| *Paenibacillus sp.* #210 | Sucrose: 100 g/L  Temperature: 40 °C  Agitation speed: 140 rpm  Time: 24 h | 1.45 | [40] |

Table S2b Comparison between FTIR values of levan produced by *Sporosarcina globispora* MTCC 4776, and other bacteria

| Peak (cm⁻¹) | *Sporosarcina globispora* MTCC 4776 | *Bacillus subtilis* ZW019 | *Bacillus siamensis* | *Bacillus megaterium* GJT321 |
| --- | --- | --- | --- | --- |
| O–H stretch | 3315 | 3304 | 3275 | 3355 |
| C–H stretch | 2933 | 2931 | 2934 | 2935 |
| Bound water | 1648 | 1644 | 1642 | 1648 |
| Glycosidic C–O–C | 1109, 1012 | 1122, 1009 | 1123, 1012 | 1129, 1011 |
| Furanose ring | 919 | 923 | 924 | 924 |
| Reference | This study | [41] | [22] | [42] |

Table S2c Comparison between ^1^H NMR peak values of levan produced by *Sporosarcina globispora* MTCC 4776, and other bacteria

| Proton assignment | *Sporosarcina globispora* MTCC 4776 | *Bacillus subtilis* ZW019 | *Bacillus siamensis* | *Bacillus megaterium* GJT321 |
| --- | --- | --- | --- | --- |
| H1a | 3.67 | 3.68 | 3.67 | 3.68 |
| H1b | 3.59 | 3.60 | 3.60 | 3.61 |
| H3 | 4.12 | 4.10 | 4.09 | 4.10 |
| H4 | 3.98 | 4.01 | 4.01 | 4.01 |
| H5 | 3.91 | 3.87 | 3.86 | 3.87 |
| H6a | 3.82 | 3.82 | 3.82 | 3.82 |
| H6b | 3.48 | 3.48 | 3.48 | 3.48 |
| References | This study | [41] | [22] | [42] |
