# Supplementary material for: Process optimization and characterization of levan from Sporosarcina globispora MTCC 4776
Source: Sci Rep. 2026 Jul 16;16:25704. doi: 10.1038/s41598-026-62960-y (PMC13482570; doi:10.1038/s41598-026-62960-y)
Supplement: Supplementary file 2 [file 41598_2026_62960_MOESM2_ESM.docx]

Fig. S1a Urease test: (a) Broth and (b) agar slant showing positive results, indicated by color change

Table S1a: EPS yield in response to different carbon source formulations

| **Media** | **A** | **B** | **C** | **D** | **E** | **F** | **G** | **H** | **I** | **J** | **Yield (g/L)** |
| --- | --- | --- | --- | --- | --- | --- | --- | --- | --- | --- | --- |
| **M1** | 10 |  |  | 0.2 |  | 0.3 | 0.02 | 0.2 |  |  | **0.33** |
| **M2** | 10 | 5 |  | 0.2 |  | 0.3 | 0.02 | 0.2 |  |  | **1.015** |
| **M3** | 10 |  | 5 | 0.2 |  | 0.3 | 0.02 | 0.2 |  |  | **0.282** |
| **M4** |  |  | 10 | 0.2 |  | 0.3 | 0.02 | 0.2 |  |  | **0** |
| **M5** |  | 10 |  | 0.2 |  | 0.3 | 0.02 | 0.2 |  |  | **1.325** |
| **M6** | 20 |  |  |  | 6 | 1.1 | 0.3 | 0.2 | 0.2 | 0.3 | **0.474** |
| **M7** | 20 | 10 |  |  | 6 | 1.1 | 0.3 | 0.2 | 0.2 | 0.3 | **0.693** |
| **M8** | 20 |  | 10 |  | 6 | 1.1 | 0.3 | 0.2 | 0.2 | 0.3 | **0.437** |
| **M9** |  |  | 20 |  | 6 | 1.1 | 0.3 | 0.2 | 0.2 | 0.3 | **0.23** |
| **M10** |  | 20 |  |  | 6 | 1.1 | 0.3 | 0.2 | 0.2 | 0.3 | **0.54** |
| **M11** |  | 20 | 20 |  | 6 | 1.1 | 0.3 | 0.2 | 0.2 | 0.3 | **0.263** |
| (% w/v) A-sucrose; B-glucose; C- fructose; D-yeast extract; E- NH₄Cl; F- K₂HPO₄; G- MgSO₄·7H₂O; H- CaCl₂·2H₂O; I- FeCl₃; J- MnSO₄·H₂O. | | | | | | | | | | | |

Table S1b: EPS yield in response to different nitrogen source formulations

| **Media** | **A** | **B** | **C** | **D** | **E** | **F** | **G** | **H** | **I** | **J** | **Yield (g/L)** |
| --- | --- | --- | --- | --- | --- | --- | --- | --- | --- | --- | --- |
| **M1** | 10 | 0.3 | 0.02 | 0.2 | 0.2 |  |  |  |  |  | **0.619** |
| **M2** | 10 | 0.3 | 0.02 | 0.2 |  | 0.2 |  |  |  |  | **0.727** |
| **M3** | 10 | 0.3 | 0.02 | 0.2 |  |  | 0.2 |  |  |  | **0.348** |
| **M4** | 10 | 0.3 | 0.02 | 0.2 |  |  |  | 0.2 |  |  | **0** |
| **M5** | 10 | 0.3 | 0.02 | 0.2 |  |  |  |  | 6 |  | **0.144** |
| **M6** | 10 | 0.3 | 0.02 | 0.2 |  |  |  |  |  | 6 | **0** |
| (% w/v) A-sucrose; B- K₂HPO₄; C- MgSO₄·7H₂O; D- CaCl₂·2H₂O; E-yeast extract; F-tryptone; G-peptone; H-casein; I- NH₄Cl; I- FeCl₃; J- (NH_4_)_2_SO_4_) | | | | | | | | | | | |

Table S1c: Coded levels, their corresponding descriptions, and actual values of process parameters used in CCD

| **Description** | **Axial low** | **Factorial low** | **Centre point** | **Factorial high** | **Axial high** |
| --- | --- | --- | --- | --- | --- |
| **Coded level** | -2 | -1 | 0 | +1 | +2 |
| **Sucrose concentration (% w/v)** | 25 | 30 | 35 | 40 | 45 |
| **Agitation speed (rpm)** | 130 | 135 | 140 | 145 | 150 |
| **Tryptone concentration (% w/v)** | 0.2 | 0.3 | 0.4 | 0.5 | 0.6 |
